# Meta-analysis: how does posterior parietal cortex contribute to reasoning?

**DOI:** 10.3389/fnhum.2014.01042

**Published:** 2015-01-21

**Authors:** Carter Wendelken

**Affiliations:** Helen Wills Neuroscience Institute, University of CaliforniaBerkeley, CA, USA

**Keywords:** deductive reasoning, posterior parietal cortex, IPL, SPL, numerical cognition, spatial cognition, meta-analysis

## Abstract

Reasoning depends on the contribution of posterior parietal cortex (PPC). But PPC is involved in many basic operations—including spatial attention, mathematical cognition, working memory, long-term memory, and language—and the nature of its contribution to reasoning is unclear. Psychological theories of the processes underlying reasoning make divergent claims about the neural systems that are likely to be involved, and better understanding the specific contribution of PPC can help to inform these theories. We set out to address several competing hypotheses, concerning the role of PPC in reasoning: (1) reasoning involves application of formal logic and is dependent on language, with PPC activation for reasoning mainly reflective of linguistic processing; (2) reasoning involves probabilistic computation and is thus dependent on numerical processing mechanisms in PPC; and (3) reasoning is built upon the representation and processing of spatial relations, and PPC activation associated with reasoning reflects spatial processing. We conducted two separate meta-analyses. First, we pooled data from our own studies of reasoning in adults, and examined activation in PPC regions of interest (ROI). Second, we conducted an automated meta-analysis using Neurosynth, in which we examined overlap between activation maps associated with reasoning and maps associated with other key functions of PPC. In both analyses, we observed reasoning-related activation concentrated in the left Inferior Parietal Lobe (IPL). Reasoning maps demonstrated the greatest overlap with mathematical cognition. Maintenance, visuospatial, and phonological processing also demonstrated some overlap with reasoning, but a large portion of the reasoning map did not overlap with the map for any other function. This evidence suggests that the PPC’s contribution to reasoning may be most closely related to its role in mathematical cognition, but that a core component of this contribution may be specific to reasoning.

## Introduction

Reasoning, the capacity to reach novel conclusions on the basis of existing premises, is among the most complex of cognitive operations. It necessarily depends on multiple underlying capacities, but the extent of this reliance on specific mechanisms is a subject of considerable debate. One possibility is that reasoning, generally or in some cases, utilizes syntactic representations of premises and application of formal logical rules (Rips, 1994; Braine and O’Brien, 1998). If this is the case, then the representations afforded by language are likely to be central to reasoning (Kertesz and McCabe, 1975; Carruthers, 2002). Another possibility is that reasoning proceeds via the use of quasi-perceptual mental models, in which case the high-level spatial and perceptual representations upon which the models are built would be critical for reasoning (Johnson-Laird, 1983, 2001). Recent work has emphasized the role of probabilistic mechanisms, in contrast to deterministic logical rule-following, in much of human reasoning (Oaksford and Chater, 2009). To the extent that reasoning proceeds via estimation and probabilistic computation, mechanisms for number processing should be critical. Of course, multiple mechanisms are possible (see e.g., Goel et al., 2000), so these theories are not mutually exclusive.

Reasoning often depends on attention to relational structure, so the mechanisms that support basic relational processing are also likely to be key. Relational representations might depend upon semantic understanding of relational terms, in which case mechanisms of semantic processing can be expected to come into play during reasoning. Alternatively, relational representations may be built upon the representation of space and spatial relationships, in which case the mechanisms of visuospatial processing may be more central to reasoning. In addition, working memory, long-term memory, and attention are all basic cognitive mechanisms that are likely to contribute to reasoning.

Many investigations of reasoning, including our own, have highlighted the role of rostrolateral prefrontal cortex (RLPFC; Christoff et al., 2001; Bunge et al., 2005; Wendelken and Bunge, 2010; Wendelken et al., 2012). In particular, these studies have shown that RLPFC contributes to second-order relational reasoning, which involves the joint consideration or integration of multiple relations and is thought to be a core component of the reasoning capacity (Gentner and Holyoak, 1997; Halford et al., 1998; Penn et al., 2008; Chuderski, 2014). However, posterior parietal cortex (PPC) is also consistently engaged during reasoning tasks (Crone et al., 2009; Eslinger et al., 2009; Watson and Chatterjee, 2012; Wendelken et al., 2012). Like RLPFC, PPC is sensitive to the need to integrate relations, but PPC is also sensitive to the number of relations considered (Crone et al., 2009) and the specificity of those relations (Wendelken and Bunge, 2010). Furthermore, there is mounting evidence from lesion studies pointing toward a critical role for PPC in reasoning. One study of left-hemisphere stroke patients revealed that performance on a matrix reasoning task was affected by damage to the inferior parietal lobe (IPL; Baldo et al., 2010). In another recent investigation, involving patients with damage to RLPFC or parietal cortex, only patients with parietal damage were significantly impaired on a transitive inference task (Waechter et al., 2013).

That PPC makes an important contribution to reasoning is apparent; but PPC is involved in numerous cognitive functions besides reasoning. To understand PPC’s contribution to reasoning, it is critical to understand how it relates to the other functions of PPC. We summarize primary functions attributed to PPC briefly here. For more extensive review of parietal function, see Grefkes and Fink (2005), Nickel and Seitz (2005), Seghier (2013), and Humphreys and Lambon Ralph (2014).

A key function of PPC is the implementation of visuospatial attention (Mesulam, 1981; Hopfinger et al., 2001; Wager et al., 2004), and of spatial processing more generally (Marshall and Fink, 2001; Husain and Nachev, 2007; Sack, 2009; Amorapanth et al., 2010). The intraparietal sulcus (IPS), which separates the inferior and superior parietal lobes, has been shown to contribute to the maintenance of spatial location information (Todd and Marois, 2004; Xu and Chun, 2006; Ackerman and Courtney, 2012). IPL, by contrast, has been implicated as a locus of spatial relational processing (Ackerman and Courtney, 2012).

PPC has also been linked to various language processes (Binder et al., 2009; Wu et al., 2012). For example, posterior IPL, angular gyrus, particularly on the left side, has been implicated as a key locus for semantic processing (Binder et al., 2009; Seghier, 2013). Moreover, just as IPS has been implicated as the locus of visuospatial maintenance, more anterior and ventral parts of IPL have been implicated in maintenance of verbal information (Paulesu et al., 1993; Awh et al., 1996; Becker et al., 1999).

In addition to its apparent role in the maintenance of both spatial and verbal information, PPC, and in particular SPL, has also been implicated in manipulation of the contents of working memory (Marshuetz et al., 2000; Wager and Smith, 2003; Wendelken et al., 2008). Moreover, PPC contributes not only to various aspects of working memory, but also to episodic memory (for review, see Berryhill and Olson, 2012). In episodic memory, parietal activation is most commonly associated with the endorsement of stimuli as having been previously encountered (Wagner et al., 2005; Nelson et al., 2013), though associations with memory encoding (e.g., Uncapher and Wagner, 2009) and memory confidence (e.g., Johnson et al., 2013) have also been noted.

Finally, though this list is by no means exhaustive, PPC is a primary contributor to mathematical cognition (Dehaene et al., 2003; Rosenberg-Lee et al., 2011). Some aspects of mathematical cognition may be linked to verbal and spatial representations within PPC (Dehaene et al., 1999). But evidence suggests that a core numerical system, localized to IPS, may be independent of these (Dehaene et al., 2003; Cohen Kadosh et al., 2005; Nieder et al., 2006).

Whether these various functions of parietal cortex on the one hand rely on shared circuitry and similar operations, or on the other hand represent separable circuits and distinct functionality, is a subject of much debate. A number of studies have sought to parcellate PPC into distinct subdivisions with differing functional roles (e.g., Nelson et al., 2010, 2013; Mars et al., 2011), while others have sought to explain apparently diverse functions in terms of a core mechanism (e.g., Bueti and Walsh, 2009; Cabeza et al., 2012).

It is possible that PPC supports reasoning through one dominant mechanism, be it numerical processing, relational representation, language, attention, working memory, or some other function; but it is also possible that different subdivisions of PPC support reasoning in different ways (see e.g., Goel, 2007; Prado et al., 2011). Regardless, understanding the way or ways in which PPC supports reasoning is critical for understanding not only the neural implementation of reasoning, but also for understanding the extent to which reasoning depends on different cognitive mechanisms.

Here, we re-examined previously collected data to better characterize the contribution of PPC to reasoning. We pursued two broad approaches. First, we examined parietal data from our own fMRI studies of deductive reasoning, all of which included a contrast between second-order and first-order relational reasoning conditions, to determine which parietal subdivisions are most selectively engaged by the higher-order reasoning condition. Second, we expanded our investigation to a much broader collection of studies to find characteristic activation patterns across PPC for reasoning as well as for a number of other parietal functions. We compared the spatial overlap of activation patterns associated with reasoning and with other cognitive functions, to determine whether or not parietal engagement during reasoning could be best understood in relation to its involvement in these other functions of parietal cortex.

## Methods

**Figure 1:**
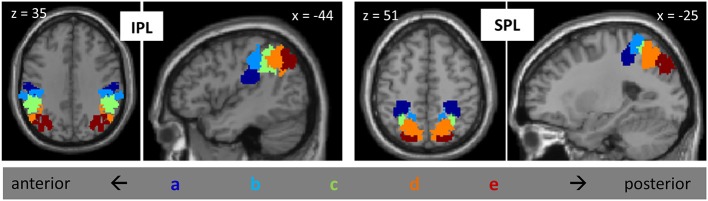
**Posterior parietal ROIs from Mars et al., 2011, including five subdivisions of IPL and five subdivisions of SPL, on the left and on the right**. Subdivisions are labeled “a” through “e”, from anterior to posterior.

All of our analyses, described below, were focused on activation patterns within PPC. Our specific parietal regions of interest (ROIs) were based on the parietal subdivisions defined in Mars et al. (2011) on the basis of tractography (Figure 1). The set of ROIs included, on each side of the brain, five subdivisions of the IPL, arrayed from anterior to posterior, and five subdivisions of the SPL, similarly arrayed from anterior to posterior; thus, there were a total of 20 parietal ROIs. For convenience, we label these regions IPLa—IPLe and SPLa—SPLe, with “a” referring to the most anterior subdivisions and “e” referring to the most posterior subdivisions. IPLa, with a center of gravity at (±49, −25, 30), is located ventral to the other IPL regions, in the parietal opercular region (Caspers et al., 2006). IPLb, with center of gravity at (±53, −32, 44), corresponds to anterior supramarginal gyrus, while IPLc, with a center of gravity at (±50, −44, 43) corresponds to posterior supramarginal gyrus. IPLd, with a center of gravity at (46, −55, 45), is located in the anterior part of the angular gyrus, and IPLe, with a center of gravity at (37, −67, 39), comprises posterior angular gyrus and the most anterior parts of the lateral occipital complex. All of these IPL regions, with the exception of IPLa, are bordered by the IPS. The anterior-most SPL region (SPLa), with a center of gravity at (30, −41, 53), was located on the anterior medial bank of the IPS. SPLb, with center of gravity at (12, −50, 63), was adjacent and medial to SPLa. SPLc, with center of gravity at (28, −55, 55), comprised the middle-to-posterior medial bank of the IPS. SPLd, with center of gravity at (19, −63, 53), was medial and posterior to SPLc. Finally, SPLe, with a center of gravity at (21, −78, 43), included the most posterior part of the medial bank of the IPS.

**Figure 2:**
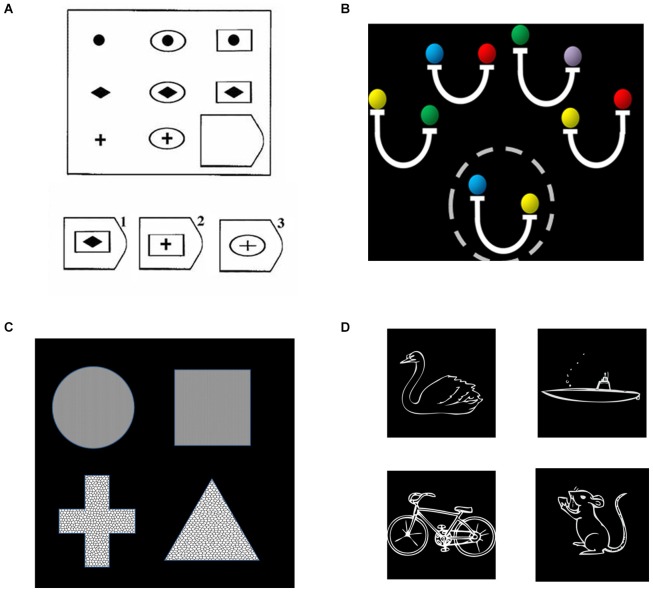
**Relational reasoning tasks, including (A) matrix reasoning; (B) transitive inference; (C) relational shape matching; and (D) relational picture matching. (A)** For relational matching, the given stimulus depicts a second-order problem, in which one must consider the relationships in both the bottom row and rightmost column to determine that the correct answer is #2. **(B)** For transitive inference, a second-order problem is shown, for which one to evaluate the validity of the probe (circled, “yellow is heavier than blue”), one must combine both the second and fourth premises (“blue is same as red” and “yellow is heavier than red”). **(C)** For the relational matching task, equivalent stimuli were used across conditions. The given stimulus is a texture match, because the top two shapes share the same texture, a shape mismatch, because neither pair share the same shape, and a relational match, because the same dimension of match (texture) is present for both the top and bottom pairs. **(D)** The semantic picture matching task follows the same logic as the relational matching task, but utilized animal vs. vehicle and land vs. water as dimensions of possible match or mismatch. The example depicts a relational match, in that the dimension of match for the top pair (land vs. water) is the same as the dimension of match for the bottom pair.

We first examined data from four different studies of relational reasoning that we have previously conducted in young adults (Bunge et al., 2009; Crone et al., 2009; Wendelken and Bunge, 2010; Wendelken et al., 2012). These deductive reasoning tasks included matrix reasoning (Raven’s Progressive Matrices), transitive inference, relational shape matching, and relational picture matching (see Figure 2). All tasks included a contrast between second-order and first-order relational reasoning conditions. For matrix reasoning, a second-order problem required consideration of both row and column to determine the correct missing element from a visuospatial array. For transitive inference, a second-order problem required combining multiple premises. The transitive inference task included problems that required consideration of directional (inequality) relations (pictured in Figure 2) as well as problems that required only consideration of non-directional (equality) relations. For both relational matching tasks, the second-order condition required participants to determine whether the top pair of stimuli matched along the same dimension as the bottom pair. All three of the above tasks involved visuospatial stimuli. By contrast, the relational picture matching task included evaluation of semantic relationships (pictured) as well as visuospatial relationships. We obtained contrast activation values for each participant, from each of the four studies, for each parietal ROI. We then submitted these contrast values to statistical analysis in SPSS, wherein we conducted an ANOVA that included parietal region, subdivision, and side as within-subjects factors and task/study as a between subjects factor.

For the broader analysis of reasoning-related activation and its relationship with other parietal functions, activation maps were obtained using Neurosynth, which provides automated meta-analyses based on Keywords (Yarkoni et al., 2011). The Neurosynth algorithm extracts clusters associated with specific key words across a large database (thousands) of neuroimaging studies. First, for a given key word (e.g., “reasoning”), it calculates frequency of appearance within an article, and identifies studies for which the key word appears at a high frequency (more than once per thousand words). Second, it automatically extracts activation coordinates from tables reported in these studies. Third, the set of coordinates extracted from studies that have been linked to a key word are submitted to multilevel kernel density analysis (MKDA) to produce activation maps (c.f. Wager et al., 2009). Finally, taking into consideration maps generated for a large number of different key words, machine learning (naïve Bayes classification) is used to estimate the likelihood that activations were associated with specific psychological terms.

In addition to “reasoning”, we utilized the following terms associated with functions of PPC: “numerical” and “calculation” for mathematical cognition, “visuospatial” and “attention” for visuospatial processing and attention, and “phonological”, “lexical”, and “semantic” for language-related processes. We also examined activation maps associated with the terms “maintenance” and “manipulation” (working memory), and “memory encoding” and “memory retrieval” (long-term memory). Table 1 gives the number of studies included for each term. For each of these terms, we obtained the reverse inference map, which displays regions that are reported more often in studies that load highly on the selected term than in studies that do not load highly on the term. In other words, the reverse inference maps display regions that are diagnostic of the term or feature. In addition, to obtain a broader representation of reasoning-related activation, we also obtained the forward inference map associated with reasoning. The forward inference map includes regions that are consistently activated in studies that load highly on the term. Thus, the forward inference reasoning map included regions that are typically activated during reasoning tasks, not all of which are particularly diagnostic of reasoning.

**Table 1:** **The number of studies included in the Neurosynth meta-analysis, for each term**.

Term	Number of studies
Reasoning	124
Visuospatial	184
Attention	1199
Memory retrieval	144
Memory encoding	101
Manipulation	204
Maintenance	224
Numerical	64
Calculation	55
Semantic	701
Phonological	260
Lexical	212

Calculations of image characteristics were done using FSL (FMRIB Software Library, Oxford Center for Functional Magnetic Resonance Imaging of the Brain). We first computed, for each term, the extent of activation within each parietal ROI. Next, we computed overlap volume between each reasoning map (forward and reverse inference) and every other feature map (reverse inference only). This was done separately for each parietal ROI. From these initial values, we computed similarity scores relating the reasoning maps to every other feature. Similarity between two maps was defined as the volume of activation in the intersection of the two maps divided by the total volume of activation in the union of the two maps; thus, non-overlapping maps would have a similarity score of 0 and maps that are the same would have a similarity score of 1. We also computed the percentage of the reasoning activation that was accounted for by each feature; this differs from the similarity score in that a large activation cluster that effectively contains the reasoning cluster, but which includes many non-reasoning voxels as well, would have a high percent-of-reasoning score but a lower similarity score.

## Results

### Posterior parietal engagement during relational reasoning

First, we sought to characterize patterns of reasoning-related activation across the posterior parietal ROIs. Figure 2A shows average percent signal change in each ROI. Notably, there was engagement across posterior IPL, and to a lesser extent across left posterior SPL. We conducted an ANOVA that included parietal region (IPL or SPL), subdivision (1–5), and hemisphere (left or right) as within-subjects factors, and task (matrix reasoning, transitive inference, shape matching, or picture matching) as a between-subjects factor. First, there was a main effect of hemisphere (*F*_(1,65)_ = 10.31, *p* = 0.002), such that activation on the left was stronger than activation on the right. Second, there was a main effect of subdivision (*F*_(4,260)_ = 17.64, *p* < 0.001). *Post hoc* tests indicated that this was driven by greater activation in the middle and posterior subdivisions (c, d, and e) relative to the anterior subdivisions (a and b; all *p*’s < 0.001). There was no main effect of region (*p* > 0.2). However, there was a significant region × subdivision interaction (*F*_(4,260)_ = 3.62, *p* = 0.007), such that increased activation for IPL vs. SPL was observed in the middle and posterior but not in the anterior subdivisions. There was also an interaction between subdivision and side (*F*_(4,12)_ = 5.06, *p* = 0.01), such that the increased activation within left vs. right PPC was strongest in the posterior subdivisions and was not present in the anterior subdivisions.

Although our purpose here was to determine commonalities across studies, we note that there were differences between these studies in terms of both the parietal subdivisions and hemisphere that were most strongly engaged, as reflected in a subdivision × task interaction (*F*_(12,260)_ = 4.62, *p* < 0.001) as well as a hemisphere × task interaction (*F*_(3,65)_ = 7.01, *p* < 0.001). Notably, the transitive inference task did not demonstrate the preferential engagement of more posterior subdivisions that was present for the other three tasks. Moreover, while three out of four tasks engaged left PPC more than right PPC, the picture matching task, which included a visuospatial component, engaged right PPC to a greater extent.

### Posterior parietal regions associated with reasoning and other tasks

**Figure 3:**
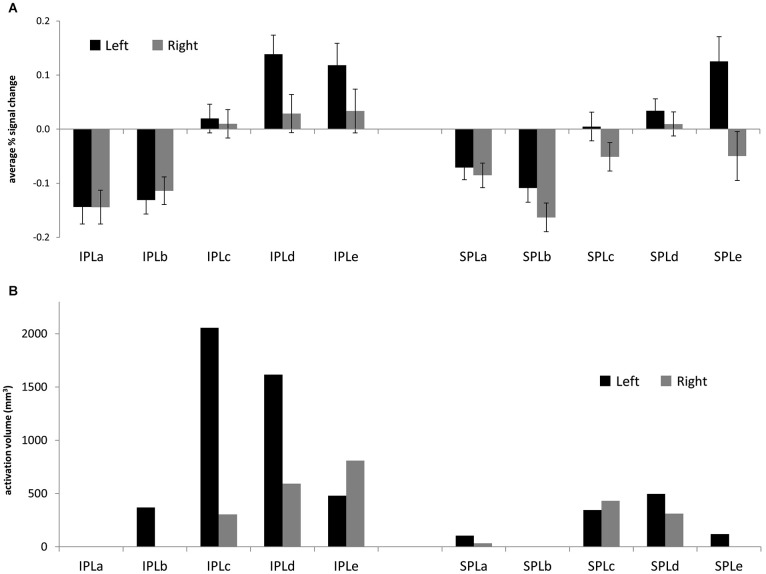
**(A)** Average % signal change for the contrast between 2nd-order and 1st-order relational reasoning, across four separate studies (matrix reasoning, transitive inference, relational matching, picture matching). Error bars are standard error of the mean across subjects. **(B)** Volume of the Neurosynth forward inference map associated with reasoning, for each posterior parietal ROI.

**Figure 4:**
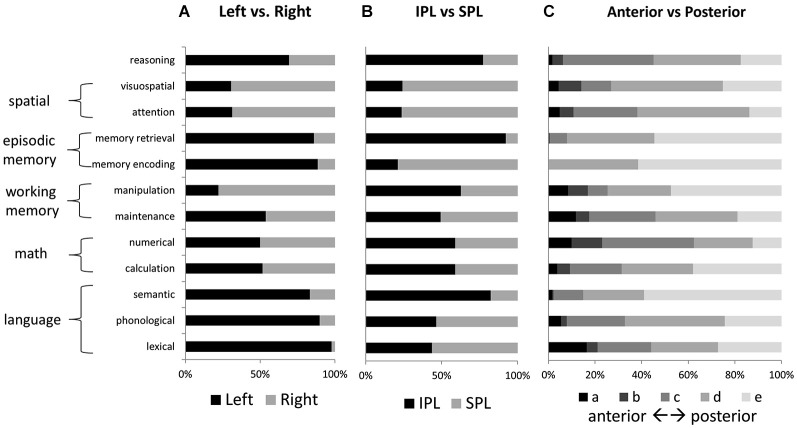
**Relative activation volumes associated with each term, for (A) left vs. right parietal cortex, and (B) IPL vs. SPL; and (C) anterior to posterior parietal cortex**. All bars add up to 100%. Terms are grouped according to the higher-level category to which they are thought to correspond.

Next, we turned to the large-scale meta-analysis and examined the extent of reasoning-related activations within each posterior parietal ROI. For the reverse inference reasoning map, which shows voxels that are most selective for reasoning, activations were almost entirely limited to the third and fourth subdivisions of left IPL (IPLc and IPLd: 51% and 42% of total active voxels, respectively;). For the forward inference map, activations were more extensive (Figure 3B), with greater volume on the left vs. right (69% left; Figure 4A) and greater volume within IPL vs. SPL (77% IPL; Figure 4B). Again, active voxels were concentrated in left IPLc and IPLd (26% and 20% of active voxels, respectively), but also spread to IPLe as well as to the more posterior subdivisions of SPL.

No other tested function demonstrated a similar concentration of active voxels within left IPLc. Memory retrieval, like reasoning, had a large share of activated voxels in left IPLd; but unlike for reasoning, memory retrieval activations were more concentrated in left IPLe. Figure 4 shows relative numbers of voxels for left vs. right PPC and for IPL vs. SPL, for each of the examined features. Language and memory activations, like reasoning, were heavily left-lateralized. In contrast, attention and visuospatial activations, as well as those for manipulation, were heavily right lateralized. Voxels associated with mathematical cognition as well as maintenance were evenly balanced across left and right. Memory retrieval and semantic processing, along with reasoning, demonstrated the strongest preferential engagement of IPL over SPL. In contrast, visuospatial processing and attention, as well as memory encoding, demonstrated notable preferential engagement of SPL.

### Similarity of reasoning to other functions in posterior parietal cortex

**Figure 5:**
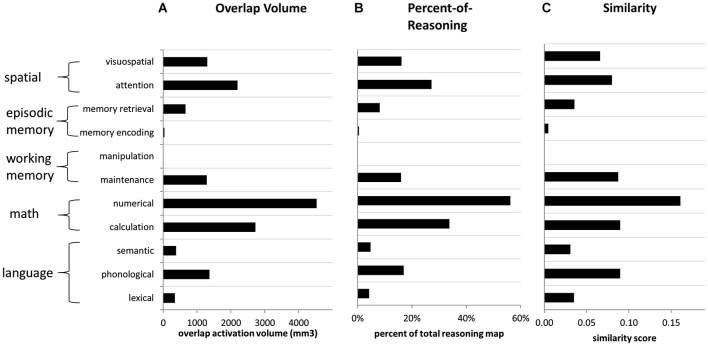
**Overlap metrics for reasoning (forward inference map) in relation to other functions (reverse inference maps). (A)** Volume of overlap with the reasoning map, for the activation maps associated with each other term. **(B)** Percent of reasoning activation from the reasoning map that overlapped with each other feature, across all parietal ROIs. **(C)** Similarity scores comparing the reasoning map to the map associated with every other feature, across all parietal ROIs. Similarity was computed as the volume of overlap divided by the total volume of activation for both features.

Our primary Neurosynth-based analysis involved examination of overlap between the activation maps associated with reasoning and those associated with other parietal functions. For each function (i.e., key word), in relation to reasoning, we examined: (1) overlap volume; (2) percentage of the reasoning volume accounted for by the overlap (“percent-of-reasoning”); and (3) percentage of the total volume (for reasoning plus the function of interest) accounted for by the overlap (“similarity”). These measures were obtained for both the forward inference and reverse inference reasoning maps. Overall results for each of the three measures are presented in Figure 5.

For the forward inference reasoning map, the feature “numerical” demonstrated the greatest overlap with reasoning across PPC. It overlapped with a large proportion (>50%) of the reasoning activation in most of the parietal ROIs that we examined, except for left IPLd, where the reasoning activation was most extensive. The numerical map also demonstrated the greatest overall similarity to the reasoning map. After numerical, the feature with the second-greatest overlap with reasoning, and also the second-highest similarity score, was calculation. Thus, the math cognition measures were most closely related to reasoning.

In addition to the math cognition features, activation maps from four other features demonstrated notable overlap with the reverse inference reasoning map: attention, visuospatial, phonological, and maintenance. Among these features, the attention and visuospatial maps demonstrated the greatest overlap with reasoning on the right side, particularly in IPLd, IPLe, and SPLc. In contrast, among these four features, the phonological map demonstrated the greatest similarity to the reasoning on the left, and overlapped with nearly 50% of the reasoning activation in left SPL. The maintenance map demonstrated a more balanced pattern of similarity to the reasoning map, across the collection of parietal ROIs. For all of the other examined features, the percent-of-reasoning scores were less than 10%.

In addition to examining the forward inference activation map associated with reasoning, we also examined overlaps for the much smaller reverse inference reasoning map. Here again, numerical demonstrated the greatest overlap with reasoning, accounting for 24% of the overall reasoning activation and 25% of its activation within left IPL. The visuospatial map overlapped with 75% of the small reasoning activation within right IPL; however, it accounted for only 3% of the overall reasoning activation. In fact, no feature other than numerical accounted for more than 10% of the reasoning activation. Thus, a large proportion of the activation related to reasoning, particularly within IPLd, appears to be distinct from the activations associated with other parietal functions.

**Figure 6:**
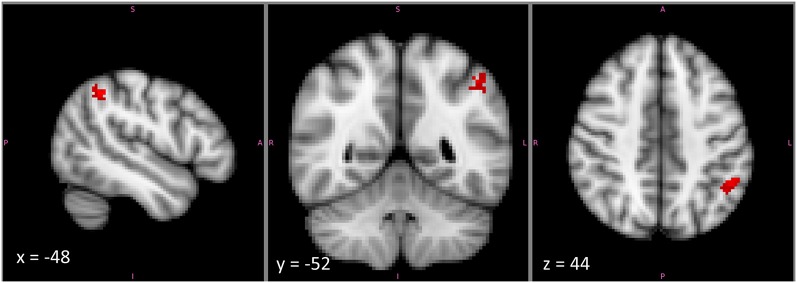
**The cluster within left mid-IPL (IPLc and IPLd) that was associated exclusively with reasoning and with no other examined term**. This cluster lies on the upper part of the ventral bank of the intraparietal sulcus. Note that the image is displayed in radiological coordinates, with left and right reversed.

Notably, there was a substantial part of the reasoning activation that did not overlap with that for any other feature. This was particularly true within left IPLd, the region that demonstrated the greatest specificity for second-order relational reasoning in our own studies. The reasoning-specific activation cluster from the Neurosynth analysis is shown in Figure 6. Although we did not formally separate dorsal and ventral subdivisions of IPL, or position along the gyrus vs. position in the depth of the IPS, it is clear from the pattern of activations that reasoning-specific activation is concentrated in dorsal IPL, on the border of the IPS but not in the sulcus. By contrast, many other functions appear to overlap more ventrally, and within the depth of the sulcus.

## Discussion

The goals of the current study were to (1) better characterize the pattern of posterior parietal engagement during reasoning; and (2) to use this information, along with information about parietal engagement in other domains, to better understand the parietal contribution to reasoning.

### Patterns of parietal engagement during reasoning

With regard to the first goal, we have obtained complementary evidence from two separate analyses that, within PPC, reasoning is most strongly associated with activation of middle to posterior IPL, and to a lesser extent with neighboring regions of middle to posterior SPL. For both analyses that we performed—of average percent signal change across four studies of relational reasoning and of activation volumes associated with reasoning in a large-scale meta-analysis—IPL demonstrated greater involvement in reasoning than did SPL, and left PPC demonstrated greater involvement than right PPC. In both analyses, the anterior-most subdivisions of IPL and SPL demonstrated no involvement in reasoning. There were some differences between the two approaches, with regard to the pattern of involvement across posterior regions: the relational reasoning tasks tended to engage the more posterior regions to a greater extent, whereas activation volumes were greatest within the middle regions for the larger-scale meta-analysis.

Both of our analyses here were focused on uncovering patterns of engagement that are common across reasoning tasks. But in addition to commonalities, we would expect, and indeed have observed, differences among different kinds of reasoning in their patterns of parietal activation. Notably, in our picture matching task, which included both visuospatial and semantic relational reasoning, we observed selectivity for higher-order visuospatial but not semantic reasoning in right PPC (Wendelken et al., 2012). In the transitive inference task, we observed stronger PPC activation for reasoning with inequalities than for reasoning with equalities, and argued that this was due to representation of the more specific inequality relationships in PPC (Wendelken and Bunge, 2010). Moreover, In a meta-analysis that directly examined different kinds of reasoning tasks, Prado et al. (2011) reported bilateral PPC activation during relational reasoning, and left PPC activation during propositional reasoning.

It is notable that the anterior subdivisions of both IPL and SPL, which were not associated with reasoning in the Neurosynth analysis, demonstrated reduced activation for second-order relative to first-order relational reasoning across our four reasoning tasks. These differences were largely driven by larger positive activations for the first-order relational task, and not by deactivation during second-order reasoning. However, this pattern of relatively reduced activation during the generally more difficult second-order reasoning condition in anterior PPC is consistent with participation this region in the default mode network (see Laird et al., 2009). Regions in the default mode network are typically deactivated during a wide spectrum of cognitively demanding tasks; thus, the deactivation in anterior PPC that we observe is likely to be non-specific to reasoning.

### The parietal contribution to reasoning

With regard to our second goal, evidence from the large-scale meta-analysis indicates clearly that the pattern of activation associated with reasoning is most closely related to that for mathematical cognition. There were also notable similarities between reasoning activations and those associated with visuospatial processing and attention, particularly on the right; between reasoning and phonological processing, particularly on the left; and between reasoning and working memory maintenance, bilaterally. These findings help to clarify the possible contributions of PPC to reasoning.

A key question is the extent to which reasoning is accomplished via mental logic and rule-following, on the one hand, or estimation and probabilistic computation, on the other. The current evidence clearly points towards the latter. Logical rule-following is posited to depend on formal language-like constructs, if not directly on linguistic representations. Although there was some similarity between reasoning and phonological activations, the overlap with mathematical cognition terms was much greater. Moreover, there was practically no parietal overlap between the reasoning map and maps associated with either lexical or semantic processing. In addition to a reliance on language-related processes, manipulation of formal logical rules can also be expected to depend heavily on processes that support manipulation in working memory. But here again, although there was some overlap between reasoning and working memory maintenance, there was practically no overlap between reasoning and manipulation. Thus, the current evidence points away from a logical rule-following as a primary mechanism for reasoning, and is more consistent with accounts that involve estimation and probabilistic computation.

An alternative explanation of the strong overlap between reasoning and math cognition is that, instead of reasoning relying on basic mathematical cognition, some types of mathematical cognition may rely on the capacity for reasoning. Indeed, advanced mathematical operations place a strong demand on reasoning, and math achievement in school is highly dependent on reasoning ability (Taub et al., 2008). It is entirely possible that some part of the overlap between reasoning and math-related activation reflects activation associated with mathematical reasoning. However, reasoning in math tasks is unlikely to fully explain the observed overlap, because the math cognition studies identified by the “numerical” and “calculation” keywords tend to involve simple tasks that put the greatest demand on basic numerical processes (e.g., magnitude estimation) and simple calculations, and put relatively less demand on reasoning.

The overlap between the reasoning map and the map associated with maintenance could reflect the importance of working memory as a component process of reasoning (Kyllonen and Christal, 1990; Salthouse, 1992). But the limited extent of this overlap argues against working memory as the main explanation for parietal engagement during reasoning. Similarly, overlap between the maps for reasoning and attention leaves open the possibility that part of the parietal activation for reasoning reflects attentional processes. Indeed, attentional processes are likely to be involved in many reasoning tasks. But here again, attention does not appear to be the primary explanation for parietal activation during reasoning.

Among potential parietal functions that we did not consider here, social cognition is worthy of mention. One recent meta-analyses highlights the tempo-parietal junction (TPJ), which includes ventral parts of IPL, as a key locus of social cognition, and points to overlap between the social cognitive function of TPJ and other parietal functions including language, memory, and attention (Carter and Huettel, 2013). However, while many of the functions that we examined do activate this ventral IPL/TPJ region, it is notable that reasoning does not, with reasoning activations mostly limited to the more dorsal parts of IPL on the border of the IPS. Notably, one class of social cognition studies—those using false belief stories—have been linked to dorsal IPL (Shurz et al., 2014). False belief studies probe the ability to reason about theory of mind. Thus, dorsal IPL activation in these studies may well be due to the reasoning demand inherent in this social cognitive task.

### Parietal specialization for reasoning?

It is notable that a large part of the activation map for reasoning—particularly in left IPL in the vicinity of the IPS—did not overlap with the maps for any of the other functions that were considered here. Of course, it is possible that some other function of PPC, not considered here, may help to explain the engagement of this region for reasoning. But the current results are at least suggestive of the possibility that this reasoning-related activation represents a fairly narrow specialization of this part of PPC for reasoning processes.

This mid-IPL region that appears as unique for reasoning in our Neurosynth analysis is similar to the IPL activations that we typically observe in studies of relational reasoning, and in particular is consistent with the region for which we reported the strongest contrast activation in our small-scale meta-analysis of relational reasoning studies. We have previously argued that RLPFC, in the frontal lobe, is specialized for second-order relational reasoning. The current results are consistent with the possibility that RLPFC may share this duty with a subregion of mid-IPL. Although direct anatomical connections between RLPFC and mid-IPL have not been reported, it is noteworthy that these two regions demonstrate strong functional connectivity during task execution (Boorman et al., 2009; Wendelken et al., 2012) and even at rest (Vincent et al., 2008).

### Limitations and future work

It is important to note several limitations in the interpretation of our findings. Our first analysis involved only a small number of studies from our lab. This approach had the advantage, over typical larger-scale meta-analyses, of allowing for extraction of whole-brain contrast images based on complete data from each study. The similarity of the tasks—each involving a contrast between second-order and first-order relational reasoning—was a key advantage that enabled this analysis. However, the fundamental similarity of these tasks, coupled with the small number, limits the generalizability of our initial findings. Beyond the fact of the small number of studies included here, and the similarity of the tasks, all of these studies drew from a similar pool of participants (UC Berkeley undergraduates) and involved similar analytical methods. Moreover, and despite the fundamental similarity of the tasks, there was variation across these studies in terms of parietal activation, and the average activation measure that we examined here only tells part of the story.

While the Neurosynth approach allowed for analysis of a much larger set of studies, individual datapoints within this analysis are much less informative and reliable. There are a number of sources of potential error in the Neurosynth approach: (1) the identification of studies by keyword will lead to both false inclusions and omissions of relevant studies; (2) the identification of coordinates within a study is done without regard to any specific contrast; (3) there is no attempt to distinguish between activations and deactivations; and (4) as with any meta-analysis, there is an inherent confirmation bias, since results that do not fit prior expectations may not be reported. Moreover, while the selection of reasoning tasks examined by Neurosynth is considerably broader than the four relational reasoning tasks examined in our initial analysis, it may still be biased towards certain types of reasoning tasks. Despite these limitations, examinations of Neurosynth results have shown them to be very much in line with those of more traditional meta-analyses. Our side-by-side examination of results from Neurosynth and from our own reasoning studies was intended partly as a validation of the Neurosynth reasoning results, though we could not validate results for other keywords in a similar manner.

Because our focus in the current study was on the contribution of PPC, our results only speak to the PPC role in reasoning, and not to the contribution of other brain regions. Thus, while we interpret the current evidence as supporting the hypothesis that mathematical or probabilistic mechanisms underlie the parietal contribution to reasoning, they do not rule out the possibility that other mechanisms (e.g., linguistic) may support reasoning through the engagement of other brain regions.

The Neurosynth-based analysis does not distinguish between reasoning tasks that are by design deductive (where conclusions follow necessarily from the premises) or inductive (where uncertainty is an explicit part of the task). It is reasonable to suppose that differences in the extent of logical rule following vs. probabilistic calculation would be present for these different kinds of reasoning. But the extent to which human reasoners employ logical rule-following to solve nominally deductive tasks, or probabilistic computation to solve nominally inductive tasks, is unclear. Much of the debate on logical rule-following vs. probabilistic computation focuses specifically on deductive reasoning (Oaksford and Chater, 2009; Khemlani and Johnson-Laird, 2012), though this debate can also apply in the case of inductive reasoning, with tools like fuzzy logic providing a possible rule-based mechanism (Smithson and Oden, 1999). Understanding how the parietal contribution to reasoning might differ as a function of deductive vs. inductive reasoning is an open question and an important follow-up to the current results.

Limitations of the approach notwithstanding, these results demonstrate the value of Neurosynth as a tool. Rigorous meta-analyses have previously characterized patterns of activation associated with reasoning (e.g., Goel, 2007; Prado et al., 2011). But Neurosynth enabled direct comparison of activation maps for reasoning and a wide range of other functions, in a manner and at a scale that would be very difficult to achieve without the automation that it provides. One of the chief ways that neuroimaging work can inform psychological theory is by telling us which functions potentially utilize the same neural circuitry. Thus, the ability to characterize a pattern of activation associated with some function of interest in terms of its overlap with many other functional patterns may emerge as a fundamental analytical tool.

## Conflict of interest statement

The author declares that the research was conducted in the absence of any commercial or financial relationships that could be construed as a potential conflict of interest.
